# Tick-Borne Pathogens and Diseases in Greece

**DOI:** 10.3390/microorganisms9081732

**Published:** 2021-08-14

**Authors:** Artemis Efstratiou, Gabriele Karanis, Panagiotis Karanis

**Affiliations:** 1 National Research Center for Protozoan Diseases, Obihiro University of Agriculture and Veterinary Medicine, Obihiro 080-8555, Japan; aefstratiou@outlook.com; 2 Orthopädische Rehabilitationsklinik, Eisenmoorbad Bad Schmiedeberg Kur GmbH, 06905 Bad Schmiedeberg, Germany; gabrielekoydl@yahoo.de; 3 Medical Faculty and University Hospital, The University of Cologne, 50923 Cologne, Germany; 4 Department of Basic and Clinical Sciences, University of Nicosia Medical School, 21 Ilia Papakyriakou, 2414 Engomi. P.O. Box 24005, Nicosia CY-1700, Cyprus

**Keywords:** tick-borne diseases, Greece, climate change

## Abstract

Tick-borne diseases (TBDs) are recognized as a serious and growing public health epidemic in Europe, and are a cause of major losses in livestock production worldwide. This review is an attempt to present a summary of results from studies conducted over the last century until the end of the year 2020 regarding ticks, tick-borne pathogens, and tick-borne diseases in Greece. We provide an overview of the tick species found in Greece, as well as the most important tick-borne pathogens (viruses, bacteria, protozoa) and corresponding diseases in circulation. We also consider prevalence data, as well as geographic and climatic conditions. Knowledge of past and current situations of TBDs, as well as an awareness of (risk) factors affecting future developments will help to find approaches to integrated tick management as part of the ‘One Health Concept’; it will assist in avoiding the possibility of hotspot disease emergencies and intra- and intercontinental transmission. Increased surveillance in Greece is required to ensure clear and effective policies for TBD control.

## 1. Introduction

Ticks are blood-feeding ectoparasites found on mammals, birds, and reptiles worldwide. Phylogenetically, they belong to the phylum Arthropoda and are divided into two large families: Ixodidae (hard ticks) and Argasidae (soft ticks), with the former being the largest and medically most important group. As opposed to nest-dwelling tick species, non-nidicolous ticks are found on vegetation, such as grasses and bushes, onto which they climb and wait for the chance to attach themselves to their prey. They feed from sucking blood from their hosts, which can be pets, farm or wild animals, and humans. Ticks are known as carriers for a wide range of pathogens, including viruses, bacteria, protozoa, and helminths [1], and therefore, numerous tick-borne diseases (TBDs) are transmitted through their bite. Overall, it is estimated that about 10% of the over 700 known hard tick species are implicated in pathogen transmission [2,3]. As such, ticks, like mosquitoes, are recognized as the main arthropod vectors of human diseases globally, and of major medical and veterinary importance.

There are four distinct stages in a tick’s life cycle (egg, larva, nymph, and adult), the duration of which varies depending on the tick type [4,5]. For each developmental stage, the tick requires nutrients that are provided by the host’s blood, as well as specific environmental conditions; usually, temperature greater than 7 °C and humidity higher than 85%. Both factors are determinants for their activity, survival, density, and diversity [6]. Consequently, these factors also determine the risk areas for TBDs, and locations of emergence are continuous remodeled by changing local environmental conditions, amongst other things due to the global climate change. Nowadays, the incidence of TBDs is on the rise worldwide [7]; diseases, such as babesiosis, ehrlichiosis, and anaplasmosis, have all shown evidence of increased prevalence and distribution in various parts of Europe, where ticks are recognized as the most widespread and prevalent of all medically significant transmitters. In anticipation of novel outbreaks of emerging vector-borne diseases, the European Union has launched the ‘Emerging and Vector-borne Diseases Programme’, in order to strengthen the EU-wide preparedness and response capabilities (https://www.ecdc.europa.eu/en/about-us/who-we-work/disease-and-laboratory-networks, accessed on 12 August 2021).

It is known that the developmental period of the tick is shortened due to the high temperatures in the environment, leading to increases in tick populations [8]. As such, the environmental conditions in Greece favor the development and proliferation of ticks. The climate is predominantly typical Mediterranean, with mild and rainy winters of a mean minimum temperature between 5–10 °C near the coasts and 0–5 °C in the mainland, and relatively warm and dry summers with mean maximum temperatures between 29 and 35 °C. The relative humidity on average ranges between 65% and 75%, with the highest in north-western Greece, and the lowest in the south, reaching peaks in December and the lowest in July–August. In climatological terms, the year can be broadly subdivided into two main seasons: the cold and rainy period lasting from mid-October until the end of March, and the warm and dry season lasting from April until September (Hellenic National Meteorological Service, www.hnms.gr, accessed on 12 August 2021). Greece, therefore, probably has a distinct population of different tick species, increasing the risk of TBDs occurrence. Furthermore, studies show an increasing trend towards dry spells and overall drier conditions in the Eastern Mediterranean region, including Greece [9,10]. Together with climatic simulations predicting a significant increase of mean maximum temperatures in the entire country [11], associated changes in tick vector distributions are likely to pose new challenges to public health. Unfortunately, research initiatives in public health in the country are often inadequate, and are focused in areas other than parasitic and vector-borne diseases. A chronic lack of funding for projects on TBDs means that an organized effort for the systematic gathering of data on ticks and TBDs in the country has not yet been undertaken, as has been the case for other EU countries. This would be a crucial first step towards the control of TBDs, which, alongside other vector-borne diseases, continue to be a major source of disease, disability, and death worldwide [12,13,14].

The present review is a systematic effort to present and analyze all available data on tick-borne pathogens and TBDs in Greece, as extracted from relevant epidemiological studies and reports published throughout the 20th century and until December 2020. The collection of data was based upon available global literature sources. The literature search used the MEDLINE/PubMed and Scopus databases, as well as available electronic data from E.U. surveillance systems, including the European Centre of Disease Prevention and Control (ECDC). The collection of data entailing this present review was also based on the use of online information from Euro Surveillance (published by ECDC). In the above electronic databases, the general terms “Greece (and) ticks”, “Greece (and) tick-borne disease”, as well as keywords of specific tick species (e.g., “Greece (and) *Ixodes ricinus*”), tick-borne pathogens (e.g., “Greece (and) tick-borne encephalitis virus”), and tick-borne diseases (e.g., “Greece (and) tick-borne encephalitis”) were applied and the listed articles critically reviewed. The collected information thus pertains to all different tick species found to be present in the country, as well as their corresponding tick-borne pathogens (in order: viruses, bacteria, and protozoa). Additionally, the specific threats that each tick-borne pathogen poses to human and livestock health are briefly addressed, along with notable cases that have been documented in the scientific literature. The aim of this work is to shed light onto the past and current epidemiological situation of TBDs and their risk in Greece, with the hope of supporting and guiding future national public health strategies for their control.

## 2. Prevalence of Pathogen-Transmitting Tick Species in Greece

To date, a total of 26 hard tick species and subspecies parasitizing domestic animals and humans in Greece have been identified. Table 1 presents all tick species that have been reported as present in the country, alongside their region of detection, the host species from which they have been isolated, and the pathogens with which they have been associated, in studies conducted in Greece. Importantly, the majority (14 out of 26) of tick species existing in Greece are known carriers of pathogens, with most of them (11 out of 14) being reported as carriers of more than one pathogen. Table 2 presents a systematic overview of the key findings on the prevalence of hard tick species parasitizing animals and/or humans in Greece, arranged in chronological order based on publication date. Data were extracted from all relevant published studies, and include the geographic location, sampling period, sampling strategy, host species sampled, number of ticks collected, and the reported tick species’ distribution.

The first—and to this day, largest—study of this kind targeted ticks found on domestic animals throughout Macedonia during 1983–1986 [15]. In total, 11,620 ticks were collected from cattle, sheep, goats, and dogs, belonging to 16 species, the most common of which was *Rhipicephalus bursa* (36.3%), a species of great importance in veterinary medicine as it is the vector of *Babesia ovis, Theileria ovis*, and *Anaplasma ovis,* which affect sheep and goats, and the vector of *Theileria equi* infecting horses.

In a country-wide study that took place during March–October 2012 and 2013, Chaligiannis et al. collected a total of 142 ticks from cattle, and 701 ticks from infected humans who had visited hospitals [19]. From the ticks found on cattle, most were identified as *Hyalomma (Hy.) dromedarii* (47.2%), *Hy. marginatum* (17%), and *Rhipicephalus (Rh.) sanguineus* (10.5%). Ten different species of human-parasitizing ticks were identified, with the most prevalent being *Rh. sanguineus* (80.17%), *Rh. turanicus* (4.85%), and *Hy. marginatum* (4.28%). Notably, the majority of ticks of the study belonged to the *Rh. sanguineus* group, which is the main vector of the bacterial pathogen *Rickettsia conorii*, while *Hy. marginatum*, the vector of the Crimean-Congo hemorrhagic fever virus, was also present.

In a third representative study, Chaligiannis et al. collected 2108 ticks from 1201 sheep and 681 goats all over the country during March–October 2012 and 2013, and 14 different tick species were identified in total, with the widest distribution belonging to *Rh. sanguineus* (64.8%), *Rh. bursa* (25.9%), and *Dermacentor (D.) marginatus* (4.1%) [20].

It is clear from the above studies on tick prevalence that a wide variety of hard tick species parasitizing farm animals and humans alike exist in Greece. Since the risk of TBDs depends not only on the presence, but also on the pathogenic load of a given tick population, studies have attempted to shed light on the pathogens present in ticks collected from farm animals and humans in Greece.

Chaligiannis et al. collected a total of 179 adult ticks from randomly sampled sheep and goats, and from dogs presented to veterinary clinics from central and northern mainland Greece during March–October 2014 [23]. The most prevalent tick species were *Rh. sanguineus* (43%), *D. marginatus* (22.3%), *Haemaphysalis (H.) parva* (20%), and *H. sulcata* (12.3%). When screened for the presence of tick-borne pathogens of human and veterinary importance, it was found that more than half (51.4%) of the ticks carried DNA from a potential pathogen. Single infections were recognized in 42 ticks (23.5%), and multiple infections in 52 ticks (29.0%). Specifically, 20.1% of ticks were found to be positive for *Anaplasma* spp., 15.6% for *Babesia* spp., 17.9% for *Coxiella burnetii,* 15.1% for *Rickettsia* spp., and 21.2% *Theileria* spp.

In another study, 64 ticks were collected from a total of 28 goats in 5 prefectures of northern Greece between February 2015 and May 2016 [21]. Ticks were identified as *Rh. bursa* (57.8%)*, D. marginatus* (17.2%)*, I. ricinus* (15.6%)*, Rh. sanguineus* (4.7%), and *H. parva* (3.1%). Known bacterial pathogens, *Rickettsia* and *Anaplasma (A.)*, were detected in 11 ticks collected from 5 of the 28 goats: *R. monacensis* was detected in six *I. ricinus* ticks (from three goats) and *R. massiliae* was detected in one *Rh. bursa* tick; *A. phagocytophilum* was detected in one *I. ricinus* tick, and *A. platys* was detected in three *I. ricinus* ticks (collected from one goat).

It is apparent that a plethora of different hard tick species and subspecies exist in the country, able to parasitize humans and/or domestic animals. Additionally, and as will be discussed later in detail in this review, studies show that the tick species present in the country carry a wide variety of tick-borne pathogens at non-negligible rates, a potential source of concern for livestock and public health. Table 3 provides an overview of all tick-borne pathogens that have been detected in ticks and/or humans in Greece according to the bibliography, together with their tick vectors, associated diseases, clinical symptoms, and prevalence, wherever information was available.

## 3. Tick-Transmitted Viruses and Diseases, and Associated Risks

### 3.1. Tick-Borne Encephalitis Virus

Tick-borne encephalitis (TBE) is one of the most dangerous viral infections of the central nervous system in humans, and occurs in Europe and many parts of Asia. The causing agent, TBE virus (TBEV) (genus Flavivirus, family Flaviviridae), is transmitted to vertebrates through the bite of infected ticks. Ticks acquire the infection while feeding as nymphs and larvae on forest-dwelling rodents, which are widespread throughout the European continent [88]. According to the latest taxonomy on flaviviruses, TBE virus has three subtypes: European, Far Eastern, and Siberian [88]. The European subtype is most often transmitted to humans by *Ixodes ricinus* ticks, the principal vector and reservoir, and *I. persulcatus* ticks [89]. The European subtype includes all known isolates from Europe, causing a typically biphasic disease with a mortality rate of 1–2% [90]. TBE frequently clinically manifests in two phases. The first phase, lasting between one and eight days, shows up with unspecific symptoms, such as fever, headache, fatigue, muscular ache, and nausea. The second phase includes neurological symptoms of meningitis and encephalitis.

#### 3.1.1. TBE Virus and Its Reservoirs

A strain of the TBE sero-complex was isolated from the brain of a newborn goat with encephalitis symptoms in northern Greece in 1969, and was named Greek Goat Encephalitis (GGE) virus [49], which is currently classified in the TBEV group. It was suggested at that time that GGE virus might represent a third subtype because it differed antigenically from all strains belonging to the types I and II of the TBE viruses that were known at that time [91]. Since its first detection, various animals farmed in northern Greece have been found seropositive for the TBE virus [50]. A strain of GGE virus nearly identical to the prototype was identified from 10 *Ixodes (I.) ricinus* ticks collected in November 2004 from permanently grazing goats in a village in Chalkidiki prefecture, northern Greece [35]. In a larger study, Pavlidou et al. collected a total of 3144 ticks from grazing goats and sheep in rural areas of 11 prefectures in northern Greece during April–July and September–December of 2003–2006 [45]. *I. ricinus* was found to be the most prevalent (46%) tick species of the study, with two pools testing positive for GGEV. Assuming that one tick per pool was positive, the frequency of infected *I. ricinus* ticks was 0.78% in Chalkidiki in 2004 and 5.26% in Kastoria in 2006. Both positive ticks were carrying GGE virus, which has been suggested to be more resistant to the dry, hot summers than other TBE virus strains. Interestingly, the positive ticks were detected in Chalkidiki and Kastoria, both regions of high TBE prevalence of antibodies in humans (5.82% and 2.43%, respectively, during 2003–2005) [48].

#### 3.1.2. Prevalence of TBE Virus in Humans

Data regarding TBE prevalence, its epidemiology, and associated risk factors in Greece are rather limited, with only three published studies (Table 4). The first evidence of TBE virus human infections was reported during an investigation during the 1927–1928 dengue epidemic, when antibodies to the TBE virus were detected in 1.8% of human serum samples [92]. Antoniadis et al. collected sera from 475 apparently healthy individuals (farmers, wood cutters, and shepherds) from throughout Greece annually from 1981 to 1988, and reported an overall 1.7% seropositivity rate [47]. Surprisingly, the area with the highest seroprevalence (13.8%) was Laconia in Peloponnese, a region where *I. ricinus* ticks have not been shown to be prevalent. TBE virus also appeared endemic in Macedonia (Imathia, Pella) and Epirus (Ioannina), areas where a high prevalence of *I. ricinus* has been well documented [15,21,45]). In an investigation during 2003–2005 throughout northern Greece and Corfu Island, 2.06% of the 921 apparently healthy individuals were found to carry IgG antibodies against TBE virus [48]. As mentioned previously, a high TBE prevalence of antibodies in humans (5.82% and 2.43%, respectively) was found in Chalkidiki and Kastoria [48], correlating to a high number of infected *I. ricinus* ticks.

All the above studies show that a flavivirus of the TBE serocomplex is circulating in Greece, yet it is not a major public health problem.

### 3.2. Crimean-Congo Hemorrhagic Fever Virus

Crimean-Congo hemorrhagic fever (CCHF) is an emerging tick-borne zoonotic disease, which occurs in parts of Africa, Asia, southeastern Europe, and the Middle East [93]. The causative agent, CCHF virus (CCHFV), belongs to the genus *Nairovirus* and it is known to be the most widespread of the tick-borne viruses important to human health [94]. CCHFV is transmitted to humans primarily by a tick bite from the genus *Hyalomma (Hy.)*, but also through direct contact with the blood or tissues of infected humans or animals [95]. CCHFV causes severe hemorrhagic fever outbreaks, with a case fatality rate of 5–40% in humans. Besides the unspecific symptoms like headaches, muscle pain, and fever two weeks following exposure, the disease is characterized by hemorrhage on the skin.

#### 3.2.1. The CCHFV and the Greek Nucleoprotein AP 92

Rangunwala et al. determined the genetic diversity in CCHFV based on data available for the S gene encoding CCHFV nucleoprotein (NP) [96]. A genetic analysis of isolates using complete sequence data identified a single isolate from Greece, AP 92, as having the highest nucleotide and amino-acid diversity (8.7%) [96]. The AP 92 CCHF virus strain differs from all of the other CCHF virus strains [97]. In Greece, it was first isolated from *Rhipicephalus bursa* ticks parasitizing goats in northern Greece, in 1975 [56].

#### 3.2.2. CCHFV Tick Vectors and Seroprevalence in Animals

The CCHF virus circulates in nature, mainly among cattle, sheep, goats, and hares, with *Hy. marginatum* ticks being considered as its main transmitter. *Hy. marginatum* has been present in 12.4% of ticks collected from domestic animals in northern Greece [18], and in 5.2% of ticks removed from humans in northeastern Greece [36]. However, in a study by Papa et al., CCHFV was detected in 2.8% of the tick pools from a total of 2000 ticks collected from livestock, with most positive pathogen carrier ticks belonging to the species *Rh. sanguineus* and *Rh. bursa*, while all *Hy. marginatum* ticks were negative [42]. Furthermore, in an isolated case during April to June 2012, a CCHFV-positive *Rh. bursa* tick was removed from a sheep in the Kastoria region of northern Greece out of a total of 95 ticks collected [33]. More recently, in a study conducted in central, western, and northern Greece, de Mera et al. found one CCHFV AP92-posititve *D. marginatus* and one *Haemaphysalis parva* tick collected from a goat and a sheep, respectively [22].

Regarding CCHF seroprevalence in animals, Schuster et al. investigated a total of 538 cattle and 81 sheep in Macedonia [98]. Antiviral immune responses were observed in 6% of cattle and in 1% of sheep, with the highest cattle seroprevalence in Chalkidiki (38%). This study demonstrated a high transmission risk to humans in specific geographical areas, which should be addressed by national and local public health authorities. Although the CCHFV strains present in Greece probably do not represent a high risk to the population, the circulation of genetically diverse strains could potentially result in the appearance of novel viral genotypes with increased pathogenicity and fitness.

#### 3.2.3. Seroprevalence of CCHFV in Humans

In total, there have been nine published studies on the human seroprevalence of CCHFV in Greece (Table 4). One of the earliest studies regarding the occurrence of CCHFV in humans in the country was conducted in northern Greece, where evidence of previous CCHF infections was found in 6% of tested adults [57]. In another study, it was reported that about 1.2% of 597 residents of northern Greece tested positive for CCHFV; however, no CCHFV infection was diagnosed in patients with symptoms resembling CCHF [30]. In a different study, Antoniadis et al. tested 3388 healthy farmers, woodcutters, and shepherds from all over Greece, of whom 1% had antibodies against CCHFV [47]. The highest seroprevalence for CCHFV was documented in Pella, in the north of the country (9.6%).

Several seroprevalence studies were undertaken in order to estimate Greek endemic areas for CCHF. Sidira et al. conducted a country-wide study between June 2009 and December 2010, sampling 1611 residents of 28 prefectures, and found an overall seroprevalence of 4.2%, with significant differences among prefectures, ranging from 0% to 27.5% [52]. The highest seroprevalence rates were observed in prefectures of central-western Greece (Grevena, Fthiotida, Thesprotia), where livestock husbandry is the main occupation of residents. This study presented an increased circulation of CCHFV in Greece, as compared to previous results from the 1980s [47]. Following the above findings, Papa et al. confirmed a high seroprevalence of CCHFV in Thesprotia, reporting a seropositivity of 14.4% from 166 residents randomly selected among persons who visited local health centers [53]. Thesprotia has well-developed livestock breeding, with high sheep, goat, and cattle population densities, and a significant relation between seropositivity and contact with sheep/goats/cattle was observed. The authors noted that infections in Greece are seemingly subclinical, related with a low or non-pathogenic CCHFV strain [53]. Another serosurvey of 207 randomly sampled permanent residents of Achaia prefecture, western Greece also found a relatively high seroprevalence of 3.4% [55], with seropositive individuals encountered in rural areas where the economy is based almost entirely on livestock farming. Research at Imathia and Pella prefectures by Sidira et al. estimated the CCHFV seroprevalence among residents to be about 1.7% and 2.9%, respectively [62]. Furthermore, in the Kastoria prefecture of northern Greece, 6% of samples collected from residents of an area where a CCHFV-infected *Rh. bursa* tick had been detected were positive [33]. This rate was similar to the rate observed previously in the whole Kastoria regional unit [52]. The circulation of this specific CCHFV lineage in Greece, especially in a region where the seroprevalence is high, together with the lack of human CCHF cases suggests a probable antigenic but non- or low-pathogenic characters for this lineage [33]. Papa et al. confirmed the seropositivity rates reported in various previous studies [52,53,54] by testing serum samples using multiple methods [59]. The results established that the CCHFV seroprevalence in Greece is high, especially in the areas where livestock husbandry is present.

The first fatal non-imported case of CCHF was recorded in northern Greece in June 2008 [51]. The causative CCHFV strain was phylogenetically similar to strains circulating in Balkan countries, Turkey, and southwest Russia [58]. Following the fatal case, Papa et al. conducted a survey using 1178 samples from the residents of 5 prefectures of northern Greece, and found a mean seropositivity of 3.1% [54], which was considerably elevated compared to the rates reported in the same areas 20 years before [47]. Introduction of the virus in this region between the years 1990 and 2010 was therefore suggested, in accordance with a considerable number of CCHF outbreaks in south-eastern Europe between 2000 and 2008, attributed to climate and environmental changes, especially mild winters and the disruption of agricultural activities [99]. Recently, in June 2018, the first imported non-lethal case of severe CCHF was reported in Greece, with a virus strain belonging to clade Europe 1 [61].

### 3.3. Tick-Borne Phleboviruses and Diseases

Viruses of the *Phlebovirus* genus (family *Bunyaviridae*) are divided into two groups: the sandfly fever virus group, transmitted by sandflies and mosquitoes, and the Uukuniemi-like viruses, transmitted by ticks [100]. Interest in tick-borne phleboviruses has increased rapidly since 2009, when two novel viruses were associated with diseases in humans: severe fever with thrombocytopenia syndrome virus (SFTSV; synonym Huaiyangshan virus, HYSV; Henan fever virus, HNFV) identified in China [101] and the Heartland virus (HRTV) identified in the United States [102]. Both viruses cause a disease characterized by fever, thrombocytopenia, leukocytopenia, and multi-organ dysfunction, with a significant mortality rate.

#### Tick-Borne Phlebovirus Prevalence in Ticks

The first attempt to detect tick-borne phleboviruses in Greece was carried out by [103], who collected 287 ticks from sheep and goats from 13 regional units of northern and central Greece. Positive tick pools consisted of *Rh. sanguineus* ticks from sheep in three regions: Pella (6.7%), Imathia (9.5%), and Ioannina (7.1%). The minimum overall tick infection rate for the areas tested was 2.1%. The sequences of the Greek phlebovirus, named Antigone virus (ANTV), form a distinct clade in tick-borne phleboviruses, differing by >40% from other known phlebovirus species. The detection of ANTV in high infection rates suggests that they are widely and densely distributed, prompting further studies to find out about their geographic distribution, genetic diversity, and probable implication in public health. Papa et al. investigated the presence of tick-borne phleboviruses from 31 ticks collected from sheep in Lesvos Island during November 2015 [25]. Phleboviral RNA was detected in 12 out of the 22 *Haemaphysalis (H.) parva* ticks. Further studies are required to determine the virus distribution in other parts of Greece and in other countries, allowing a more in-depth analysis of the ecology and epidemiology of the newly detected virus.

## 4. Tick-Transmitted Bacteria and Diseases, and Associated Tick Species

### 4.1. Spotted Fever Group Rickettsioses

Rickettsioses are a group of diseases caused by *Rickettsia (R.)* species of Gram-negative, obligate intracellular bacteria. Numerous *Rickettsia* species, pathogenic to humans, occur worldwide, where several tick species act as their vectors. Rickettsioses are classified into two main groups: the spotted fever group (SFG), transmitted by ticks or mites, and the typhus group, mainly transmitted by lice or fleas. SFG *rickettsiae* (SFGR) are the bacteria that cause the human tick-borne disease spotted fever, which typically presents on the skin with papular exanthem, eschar, and spots, combined with high fever, joint and muscle pain, headache, and photophobia. The human diseases that belong to the SFG are the Mediterranean spotted fever (MSF) caused by *R. conorii*, tick-borne lymphadenopathy (TIBOLA) caused by *R. aeschlimannii* and *R. slovaca*, and lymphangitis-associated rickettsiosis (LAR) caused by *R. sibirica mongolotimonae*, all described below and presented in Table 3.

#### 4.1.1. Tick Vectors for SFGR

There have been some studies published regarding the occurrence of SFGR tick vectors in Greece. Psaroulaki et al. conducted a study in Fokida, central Greece in summer 2008, collecting a total of 439 ticks from goats, sheep, and dogs [44]. The infection rate by *Rickettsiae* (including *R. massiliae*, *R. conorii*, and *R. rhipicephali*) was 2.4% among *Rh. sanguineus* ticks and 1.4% among *Rh. bursa* ticks. Psaroulaki et al. examined 1848 ticks from various animals in Cephalonia Island, and detected 4 different SFGR from 10 infected ticks: *R. conorii* was detected in three *Rh. sanguineus* ticks from dogs; *R. massiliae* was detected in four *Rh. turanicus* ticks from cattle, sheep, and goats; *R. rhipicephali* was detected in two *Rh. sanguineus* ticks collected from dogs; and—for the first time in Greece—*R. aeschlimannii* was detected in a *Hy. anatolicum excavatum* tick, parasitizing on sheep [17]. Another study conducted in 2018 reported the presence of novel genetic variants of *R. hoogstraalii* in *Haemaphysalis sulcata* and *H. parva* ticks from goat and sheep, and *R. raoultii* in *D. marginatus* ticks from goat were reported in central and northern Greece [46].

#### 4.1.2. The Rickettsia Infectious Disease: Mediterranean Spotted Fever

Mediterranean spotted fever (MSF), one of the oldest known spotted fever group rickettsioses, is endemic in countries in and around the Mediterranean and is caused by *Rickettsia (R.) conorii* [104]. Typical clinical symptoms include high fever, myalgia, cervical lymphadenopathy, headache, and a generalized maculopapular rash. Neurological manifestations have been reported in up to 28% of patients [105]; however, the effects on the peripheral nervous system are considered extremely rare. *Rh. sanguineus*, the brown dog tick, is regarded as the main vector and reservoir of *R. conorii* [104,106]. Greece is known to be endemic for MSF and cases have been reported as early as the 1930s [107]. Sero-epidemiological studies have been undertaken in healthy populations all over Greece since 1990, and the results are presented below.

Only four published studies have examined the prevalence of MSF among the Greek population (Table 4). Antoniou et al. studied the seroprevalence and incidence of *R. conorii* in two villages in Crete, southern Greece [65]. In total, 838 blood samples from a total of 419 people were collected in 1985 and 1987. Seroprevalence was 5.6% in one village and then 2.2% after two years, and 0.5% and then 0% in the other. In a follow-up seroepidemiological study among 238 residents in one of the two villages, seroprevalence for *R. conorii* was calculated at 7.6% in 1998, increased from previous years [67].

A single study was conducted in central Greece in 1991, where a high seroprevalence of 45.3% was reported among 254 individuals from three rural villages in Fokida, central Greece [66]. Regarding the northern parts of the country, Alexiou-Daniel et al. examined 1584 serum samples from the residents of 14 prefectures during April to October 2000, and reported a mean seroprevalence of 7.9% to *R. conorii*, with the highest rates being observed in Trikala (20%), Grevena (14%), and Serres (14%) [68].

The first isolation of *R. conorii* found in a human sample in Greece was from a patient in Crete in 2002 [26]. Further cases of MSF were reported in southeastern Crete between 2000 and 2003 [69]. The cases were reported between the months May to July, which coincide with the peak period for the population of adult tick, *Rh. sanguineus*, and larvae and nymphs are active during the summer months. Contact with ticks, specifically ticks parasitizing on sheep and goats, was identified as the main risk factor. Although most cases do not present serious clinical symptoms, severe cases of MSF have been described over the years. Tzavella et al. reported a case in northern Greece with a severe form of MSF and rapid neurological deterioration, but recovery was prompt after administration of the broad-spectrum antibiotic doxycycline [70]. A fatal case of MSF was reported in a patient engaged in agricultural activities in northeastern Greece in August 2008 [64].

After this fatal case, Papa et al. collected and identified 537 ticks from humans of the same region [36]. Over 80% of the ticks were *Rh. sanguineus*. The authors suggested that the increased aggressiveness of *Rh. sanguineus* was probably related to the weather conditions, with the mean temperatures being higher than usual with minimal rainfall. Tzanetakos et al. reported the first case of a patient with a *R. conorii* infection who developed acute demyelinating motor sensory neuropathy and showed impaired fine-tune coordination of hand movements and muscle weakness, before being successfully treated with antibiotics [71].

#### 4.1.3. The Rickettsia Infectious Disease: Tick-Borne Lymphadenopathy

The bacterium *Rickettsia (R.) slovaca*, belonging to the group of tick-borne SFGR, is the causative agent of tick-borne lymphadenopathy (TIBOLA). The first laboratory-confirmed human case occurred in France in 1997 [106], and *R. slovaca* infections have since been reported in humans from several countries in Europe [108,109,110,111]. (A key clinical sign of the disease is enlargement of the regional lymph nodes following the tick bite, which is the reason for its name, tick-borne lymphadenopathy (TIBOLA), and *Dermacentor*-borne necrosis-erythema lymphadenopathy [112,113], due to *Dermacentor* (*D.*) spp. being the main vector of *R. slovaca*.

*R. slovaca* was first reported in Greece in 2010, in an examination of 703 ticks collected from goats, sheep, and dogs in Chalkidiki prefecture. *R. slovaca* was detected in a pool of *Rh. bursa* ticks collected from goats in June 2006 [40]. A year later, the first case of *R. slovaca* infection was reported, when a patient in Corfu Island developed high fever, malaise, and a maculopapular rash developed on the face, thorax, and extremities [73].

#### 4.1.4. Lymphangitis-Associated Rickettsiosis and Other SFG Rickettsia Infections in Humans

Lymphangitis-associated rickettsiosis (LAR) manifests with symptoms, such as fever, maculopapular rash, inoculation eschar, enlarged regional lymph nodes, and lymphangitis. LAR is caused by the bacteria *Rickettsia (R.) sibirica mongolotimonae*, which was reported for the first time in Greece in 2002, when it was detected in a patient in Crete and in the parasitizing *Hyalomma anatolicum excavatum* tick [31], which was also the first description of an SFGR in *Hyalomma* ticks. In 2010, a third SFG *Rickettsia* infection, caused by *R. aeschlimanni,* was reported in Crete and became the first human case of *R. aeschlimannii* infection in Europe [72]. The tick specimen removed from the patient was identified as *Rh. (Rhipicephalus) turanicus*, a species previously reported as infected with *R. aeschlimannii* in Spain [114]. More human infections with *R. conorii, R. aeschlimannii, R. sibirica mongolotimonae,* as well as a new *Rickettsia* species (originally described in Cyprus by Sandalakis et al. [115]) were diagnosed in patients in Crete [32], with three ticks (two *Rh. turanicus* and one *Hy. anatolicum excavatum)* being removed from three patients and matching their diagnosed SFGR. *R. massiliae* had previously been isolated from a *Rh. sanguineus* tick in Fokida [63]. This was the fifth case of a human *R. massiliae* infection reported in the literature, after the reported cases in Italy, Argentina, and France [116,117,118,119]. Finally, during 2008–2009, a period of particularly increased tick aggressiveness in north-eastern Greece, five *Rickettsiae* species were identified in 15% of the ticks removed from people who had sought medical advice: *R. aeschlimannii, R. massiliae,* and for the first time in Greece, *R. africae, R. monacensis*, and *Candidatus R. barbariae* [38].

### 4.2. Anaplasma and Human Granulocytic Anaplasmosis

Human granulocytic anaplasmosis (HGA) is caused by the bacterium *Anaplasma (A.) phagocytophilum,* which is primarily transmitted by *I. ricinus*. *A. phagocytophilum* infects the white blood cells and causes symptoms, such as fever, headaches, general joint and muscle pain (myalgia), and fatigue. HGA was first described in 1994 in the US [120] and has since been reported in many countries in Europe [121,122,123]; however, cases have rarely been reported in Greece.

HGA seroprevalence studies among the Greek population have been rare (Table 4). Alexiou-Daniel et al. was the first to conduct a serological survey focusing on the prevalence of HGA in Greece [77]. In total, 300 healthy human individuals, mostly farmers, from rural areas of northern Greece were tested. The overall prevalence was 7.3%, proving that HGA cases exist but are likely under-diagnosed. The first molecular evidence for the presence of *A. phagocytophilum* in *I. ricinus* ticks in the country was provided by Kachrimanidou et al., who, out of 405 examined *I. ricinus* ticks collected from goats in northern Greece in June 2006, detected four tick pools as positive [39], with the strain shared a 95% similarity with those from human cases in Crete Island previously reported [74]. In southern Greece, the seroprevalence among 496 healthy blood donors from Crete was 21.4%, and was attributed to frequent contact with animals, although no significant difference was observed among people with no contact and people with possible contact with the bacterium [76]. The first cases of severe HGA in Greece were also reported in Crete, in a study involving six patients with acute symptoms for the *A. phagocytophilum* infection [74]). Symptoms included fever, chills, maculo-papular rash of the trunk, headache, malaise, splenomegaly, cervical lymphadenopathy, and gastrointestinal disturbances. The first fatal case of HGA was reported in Athens, with a man showing symptoms of fever, sore throat, irritability, and insomnia, likely exposed to an *A. phagocytophilum*-infected tick while gardening [75]. The authors noted that this case may serve as evidence of the emergence of HGA in new geographic areas and given the presence of competent vectors. Additional cases of human anaplasmosis are therefore expected to occur in the country.

Besides humans, *A. phagocytophilum* can cause fever in ruminants and granulocytic anaplasmosis in horses and dogs. A clinical case of ovine anaplasmosis, caused by *A. phagocytophilum,* was reported for the first time in 2011 in a ram in Thessaloniki in northern Greece parasitized by *Ixodes* spp. ticks [78].

### 4.3. Borrelia and Associated Diseases

*Borrelia* (*B.*) is a genus of Gram-negative bacteria from the group of spirochetes, and includes arthropod-borne bacteria that are divided into several groups: the Lyme disease (LD) group of spirochetes, the relapsing fever (RF) group, and reptile (REP)- and echidna-associated species [29]. Various *Borrelia* species are important pathogens for humans and animals, causing infectious diseases called borreliosis. The *Borrelia* reservoir includes small rodents, such as rats and mice, from which they are transferred to different organisms using vectors, such as ticks or lice. Hard ticks of the *Ixodidae* family are common vectors of *Borrelia* bacteria. The most important *Borrelia* species transmitted by ticks are discussed below.

#### 4.3.1. Lyme Disease (Lyme Borreliosis)

All *Borrelia* species that cause Lyme disease (Lyme borreliosis) are referred to collectively as *B. burgdorferi sensu lato*, while *B. burgdorferi* itself is specified as *B. burgdorferi sensu stricto*. Lyme borreliosis is a major public health concern in Europe and North America [124]. Clinically, it may manifest multi-systemically, with the most common symptom being *Erythema migrans*, followed by neurological complications, joint and skin involvement, *Borrelia lymphocytoma*, as well as cardiovascular and renal complications.

A study by Strnad et al. reported an increase in the average densities and activities of questing ticks in parts of the European *I. ricinus* populations [124]. The distributional area of *I. ricinus* appears to be steadily shifting toward higher altitudes and latitudes, a result of climate change and its effect on vertebrate host distribution, population dynamics, and vegetation [124]. In the systematic literature study by Strnad et al., analyzing the Europe-wide prevalence of *B. burgdorferi sensu lato* in *Ixodes ricinus* ticks, there is high prevalence of ticks in western and central Europe [124]. There is only one report related to *Borrelia* in Greece (Table 4), analyzing the seroprevalence of Lyme disease in the Greek population [79]. Anti-*Borrelia burgdorferi* antibodies were detected from the blood samples of 1100 Greek male navy recruits, with a rate of 3.27%. Since *B. burgdorferi* is transmitted by *I. ricinus* ticks, which occur rarely in Greece, it was expected that publications related to *Borrelia* in ticks are rare in Greece.

#### 4.3.2. Tick-Borne Relapsing Fever Borrelia and the Diseases

Relapsing fever is the generic term for two bacterial infectious diseases: louse-borne relapsing fever (LBRF), caused mainly by *Borrelia (B.) recurrentis*, and tick-borne relapsing fever (TBRF), caused mainly by *B. duttoni*. The infections are characterized by recurrent fever, associated with recurrent spirochetemia, leading to vessel damage and organ necrosis. The occurrence of TBRF is linked to the presence of the transmitting tick species of the genus *Ornithodoros* [125].

There is limited literature for the presence of TBRF in Europe, with no reports from Greece [126]. However, an absence of literature does not necessarily mean an absence of *Borrelia* in Greece. For example, *B. miyamotoi* has been recently discovered as a relapsing fever *Borrelia* group spirochete [126], which is transmitted by the same *Ixodid* species transmitting Lyme disease.

#### 4.3.3. Another Borrelia Clade: Borrelia Turcica

The prevalence of another *Borrelia* clade was recently investigated in Greece and Turkey [28,29]. *B. turcica*, vectored by *Hy. aegyptium* (originally isolated from *Hyalomma aegyptium* in Istanbul [127]), is hosted by tortoises of the genus *Testudo,* and is of unknown pathogenicity for humans. Concerning the prevalence of *B. turcica* in Greece, tick collection was carried out from wild tortoises of the species *T. graeca* and *T. hermanni* from northern Greece in the spring of 2000 and 2017 [29]. Feeding ticks were found on *T. graeca* only and were identified as *Hy. aegyptium. Borrelia* prevalence was 38.6% in 2000, and 64.4% in 2017. Phylogenetic analysis showed that the investigated *B. turcica* samples were divergent from Lyme disease and relapsing fever species. Comparing the Greek and Turkish samples’ structure, no significant differences were found, so a question is raised about the evolution or spatial spread of this species considering the large geographic distance between collection sites [29]. As ticks do not move actively over long distances, their geographical distribution is linked to the migration of their hosts [29].

## 5. Tick-Transmitted Protozoa

### 5.1. Piroplasms and the Diseases Babesiosis and Theileriosis

Piroplasms are protozoan parasites of the phylum Apicomplexa, and comprise the tick-transmitted genera *Babesia* (B.) and *Theileria (T.)*. The disease babesiosis, caused by the protozoa *Babesia*, affects domestic and wild animals, and can cause enormous economic loss in the case of endemic outbreaks [128]. Ticks of the genus *Ixodidae,* mainly *Ixodes ricinus* in Europe, transmit *Babesia* to different vertebrates (e.g., cattle, sheep, and goats) and sometimes humans [128]. In Greece, the most common *Babesia* species that infect cattle are *Babesia bovis*, *B. bigemina*, and *B. major*, while sheep and goats are mainly parasitized by *B. ovis*. The protozoa affect the red blood cells, which are destroyed (hemolysis), resulting in anemia. Babesiosis presents with malaria-like symptoms, initially nausea, loss of appetite, tiredness, or lethargy, then high fever, muscle, and headache, which can occur alternately severely and in phases, for weeks to years, if untreated [129].

Theileriosis is an acute to subacute, highly feverish infectious disease in cattle. It is caused by *Theileria* parasites, which invade white and red blood cells, resulting in hemolysis and sometimes death. Cows during calving and young calves are at most risk. There are four different forms of theileriosis: coast fever (in eastern and southern Africa), tropical or Mediterranean theileriosis (in northern Africa, Asia, and southern Europe) [130], corridor disease (in Africa), and benign theileriosis (worldwide) [131]. The parasites *T. orientalis* and *T. annulata* infect cattle, while sheep and goats act as a host for *T. ovis*. 

#### 5.1.1. Prevalence of Piroplasms in Cattle

During the early 20th century in Greece, reports indicate that four piroplasm species, namely *Theileria (T.) orientalis, T. annulata, Babesia (B.) bigemina,* and *B. bovis*, were present [81,132,133,134]. Specifically, regions of northern Greece were considered to be endemic for piroplasmosis, particularly theileriosis [81,87]. In 1942, a fatal epidemic of tropical theileriosis in cattle was described in a village of Macedonia [135]. Theileriosis was reported mainly in imported cattle, seemingly more severely affected than local animals [136,137]. Infections with *B. bovis* constituted the most common babesiosis in cattle, with symptoms including high fever, anemia, and anorexia, whereas *B. bigemina* was less frequently observed [81].

In the first large-scale study on the prevalence of *Babesia* and *Theileria* in Greece, 602 cattle serum samples were collected from 33 localities in Macedonia during 1984–1986 [80]; 41.4% of cattle were found positive to *T. orientalis*, 2% to *T. annulata*, 21.6% to *B. bovis*, 15.2% to *B. bigemina*, and 5.1% to *B. major*. Surprisingly, there was no evidence for the occurrence of *B. divergens*, even though *l. ricinus* adult ticks, its main vector [138], have been found on cattle in the region [15]. Furthermore, *B. major* is rare in Macedonia, despite the previously established presence of its vector *H. punctata* in the locality [139]. Despite its high prevalence, *T. orientalis* does not appear to be pathogenic to cattle in Macedonia in northern Greece [80]. All proven vectors of *T. orientalis* belong to the genus *Haemaphysalis* [130]. During sampling, it appeared that there are no immature ticks in cattle [83]. *Theileria* spp. are only transmitted trans-stadially in cattle by larvae and nymphs for the accomplishment of this parasite’s life cycle. In Greece, it is possible that another tick species could serve as a vector of *T. orientalis*. The wide geographical distribution of the parasite and its high prevalence in animals indicates that the vector must be abundant, possibly *Rh. bursa*, a monotropic two-host species present throughout Macedonia [15]. Furthermore, despite the endemic status of *T. annulata* in the first half of the previous century, the incidence of tropical theileriosis in Macedonia has since significantly decreased [80]. This has been attributed to the extension of big cities and the tourist development on the coasts in the second half of the century, which has limited the grasslands available for the cattle [80]. The climate in the northern Greek regions is also not favorable for *Hyalomma detritum detritum*, the vector of *T. annulata* in southern Europe.

#### 5.1.2. Prevalence of Piroplasms in Sheep and Goats

The pathogens *Babesia (B.) ovis*, *B. motasi*, and *B. crassa* are the causes of ovine babesiosis [140], which is characterized by apathy, fever, anemia, jaundice, hemoglobinuria, and even death. In Greece, the most common *Babesia* isolate from sheep and goats is *B. ovis*, though only few relevant studies have been conducted. At the beginning of the previous century, Cardamatis reported the presence of *B. ovis* in the blood of an ewe from Thessalia [132]. Miaoulis reported the co-existence of anthrax and *B. ovis* infection in a herd of sheep [141]. Stylianopoulos and Ananiades described ovine babesiosis in imported sheep in northern Greece [81]. Cardassis and Margaritis also reported an enzootic case of what was assumed to be *Theileria ovis* in three goat herds in Macedonia [87].

The study by Papadopoulos et al. during 1984–1985 regarding the seroprevalence of *Babesia* and *Theileria* in sheep and goats in Macedonia reported that 52.1% of 721 sheep and 36.4% of 487 goats were positive for *B. ovis*, while 24.6% and 0.6% were positive for *T. ovis,* respectively [83]. In a study of 124 sheep and 32 goats from Thessaly and Epirus during August–October of 2002, the prevalence of *B. ovis* was estimated at 17.4% for sheep and 9.4% for goats [82]. These studies demonstrate the broad dispersal of *Babesia* among the Greek population of small ruminants. More recently, Giadinis et al. diagnosed sheep in northern Greece to be suffering from a hemolytic crisis with babesiosis [142], caused by a *Babesia lengau*-like organism. *B. lengau* was isolated from asymptomatic cheetahs in South Africa and has not been proven to cause disease in any animal species or human beings [143]. Regarding the occurrence of *Theileria* in small ruminants in Greece, there was only one report referring to an outbreak in three herds of goats in Macedonia in February 1959 [87], which was, at the time, ascribed to *T. ovis*. However, as *T. ovis* is known to be non-pathogenic, the severity of that outbreak (85% mortality) suggested infections due to *T. lestoquardi*, which is a malignant *Theileria* species of domestic sheep and goats [131].

#### 5.1.3. Equine Piroplasmosis-Associated Species and Their Prevalence

Equine piroplasmosis is caused by *B. caballi* and *T. equi* (formerly known as *Babesia equi*), which infect horses, mules, donkeys, ponies, and zebras through the bite of an *Ixodid* tick. The clinical signs are often non-specific and the disease can easily be confused with other similar hemolytic conditions presenting fever, anemia, and jaundice. Equine piroplasmosis is present in most tropical and sub-tropical regions of the world and is endemic in parts of Asia, South America, Africa, and Europe, especially France [144], Portugal [145], Spain [146], Italy [147], and Turkey [148].

A small number of studies have documented the occurrence of *T. equi* and *B. caballi* in Greece. Sporozoites of *B. caballi* have been detected in *Rh. sanguineus* ticks and *T. equi* has been found in *Hy. plumbeum* [34]. Kouam et al. tested 544 randomly selected, clinically healthy equids from all over mainland Greece during 2007–2008 and found a seroprevalence of 11% and 2.2% for *T. equi* and *B. caballi*, respectively [84]. The region with the highest prevalence for both parasites was Thessaly (38.8% for *T. equi* and 6.1% for *B. caballi*), while Macedonia had the lowest prevalence (6.6% for *T. equi* and 4.4% for *B. caballi*). Rates were significantly higher in mules than in horses for both parasites, which might be due to the extended exposure of mules to grassland. The enzootic status of equine piroplasmosis in Greece was supported by a large-scale study of 7872 equines from all over Greece that took place between 2001 and 2008, which found 12.9% seropositivity for *T. equi* and 1.3% for *B. caballi,* with positive samples in 39 out of the 41 prefectures [85].

Regarding the equine piroplasm genotypes occurring in the country, Kouam et al. identified two *Theileria* (*T. equi* and *T. equi*-like) species and one *Babesia* (*B. caballi*-like) genotype from equid samples all over mainland Greece [149]. Both *Theileria* and *Babesia* genotypes were detected in horses, while only *Theileria* genotypes were detected in mules and ponies. The prevalence of *Theileria* was much higher (44%) than in the previous report [84], which was attributed to the more sensitive technique used. This finding reinforces the observation that in the Mediterranean region, equine *Theileria* infections are more frequently diagnosed than *Babesia* infections [145,150,151]. A cross-sectional survey on the spatial distribution of equine piroplasms found that clusters occurred in Macedonia and Thessaly, with a high probability of the presence of both *Theileria* and *Babesia* species [86]. Macedonia and Thessaly are characterized by equine populations of a considerable size, with 27% and 10% of the total equine population of Greece, respectively [152].

### 5.2. Tick-Borne Pathogens in Dogs and Cats

Several studies have focused on the situation regarding ticks and TBDs in dogs and cats in Greece.

#### 5.2.1. Tick-Borne Pathogens in Dogs

In a study of ticks collected from 249 dogs admitted to veterinary clinics in Thessaloniki, Papazahariadou et al. identified the vast majority (89.3%) as *Rh. sanguineus*, and the remaining as *Rh. Turanicus* [43]. The high prevalence of *Rh. sanguineus* is concerning, as this species is the vector of the obligate intracellular bacterium *Ehrlichia canis,* the agent for canine monocytic ehrlichiosis (CME). Indeed, multiple cases of CME have been reported in dogs in Greece [153,154,155].

Another study by Latrofa et al. reported that 50% of 310 domestic dogs from six provinces of central and northern Greece were infested by ticks (mainly *Rh. sanguineus, Haemaphysalis parva*, *Rh. turanicus*, and *Haemaphysalis concinna*) [24]. Out of the 757 ticks collected, more than one in 10 were positive for at least one microorganism, including *Cercopithi filariabainae*, *Hepatozoon canis*, *Rickettsia hoogstraalii*, *Hepatozoon felis*, *Rickettsia massiliae*, *T. ovis*, and *Anaplasma platys* [24]. Moraga-Fernández et al. found that 3 ticks, all belonging to the *Rh. sanguineus* species, out of the 79 ticks collected during March and October 2014 from dogs in central and northern Greece were infected with *R. massiliae* [46]. In a country-wide seroprevalence study of canine vector-borne diseases, of 1000 healthy and randomly selected dogs sampled during 2016, a seroprevalence of 12.5% for *Ehrlichia* spp. and 6.2% for *Anaplasma* spp. was reported, with a single dog testing positive for *B. burgdorferi* [156]. Athanasiou et al. analyzed the blood samples of 2620 clinically healthy dogs from in seven regions of central and northern mainland Greece for the detection of, among others, *E. canis* and *B. burgdorferi* [157]. The seroprevalence was 12.25% and 2.23%, respectively, while double- and triple-pathogen seropositivity was common.

Kostopoulou et al. reported that out of a total of 1154 randomly sampled dogs of different lifestyles from four Greek islands (Crete and Leros in the Aegean Sea, and Zakynthos and Paxoi in the Ionian Sea), 7.5% were found seropositive for *Ehrlichia canis*, with an obvious tendency of a higher prevalence to the east and no cases on the Ionian Sea islands [158]. In total, 2.3% of the dogs were positive for *Anaplasma* spp., with the highest prevalence on Crete. The high positivity for *A. phagocytophilum*, especially in Crete, combined with the fact that it has been previously reported from *Rh. bursa* ticks in Greece, as well as from *Rhipicephalus* spp. ticks from other areas, indicate that there is a potential also for these ticks to play a role in the transmission of *A. phagocytophilum*.

Lastly, a group of rather neglected tick-transmitted filarioids of the genus *Cercopithifilaria* infesting the skin of dogs were shown to be surprisingly distributed in canine populations of Europe [159]. The first report of *Cercopithifilaria* spp. I in Greece showed an infection rate of 4.3% among 23 skin samples from dog from Thraki, while higher infection rates were observed in southern Italy and central Spain with 12–13.3% and 21.6%, respectively [160].

#### 5.2.2. Tick-Borne Pathogens in Cats

With regards to cats, a single publication was found, which investigated the distribution of various pathogens in 150 stray and free-roaming felines living in four regions of Greece (Crete, Mykonos, Skopelos, and Athens), and reported that 43.2% were positive for *Rickettsia* spp. [161].

### 5.3. Tick-Borne Pathogens in Wild Birds

Wild birds are of special interest in the context of tick-borne diseases (TBDs), as they are common hosts of ticks [162]. During seasonal migrations, birds that cover long distances over a short time across national and intercontinental, even transhemispheric, borders, can introduce tick and pathogen species in areas where they have never occurred before. The regionally different tick infestation of birds depends on the rhythm of the seasonal activity of ticks and the rhythm of bird migration, which are also determined by the climate and environmental factors [163]. The presence and species composition of ticks on birds depends on the migrating and feeding behavior of the hosts and on the species of ticks occurring in habitats where birds spend the breeding season and the wintering period and/or where they stop during seasonal flights. Additionally, at low ambient temperatures, the tick metabolism and feeding dynamics are slowed down, which promotes longer attachment of ticks to birds [163].

Concerning specific data from Greece, there is very limited information about ticks on birds in the country. A study by Diakou et al. reported that 2% of the 403 wild resident birds examined in northern Greece were infested with ticks of the species *Ixodes frontalis, I. acuminatus* (reported for the first time in Greece), *Hy. marginatum, Hy. Aegyptium*, and *Hyalloma* spp [27]. Four ticks were positive for *Rickettsiae* (*R. aeschlimannii* in *Hy. aegyptium* and *Rickettsia* spp. in *I. frontalis*, *Hy. Aegyptium*, and *Hy. marginatum*). All ticks were negative for *Borrelia* spp., even though *Borrelia* is the tick-borne pathogen with the highest prevalence in ticks removed from captured birds in Europe [163].

## 6. Discussion

The clinical management of tick bites in humans is part of the daily routine of the medical (hospital) outpatient clinic, especially in certain peak times depending on the seasons or climatic conditions. Insufficient or wrong treatment can lead to severe diseases with long-term consequences, including disabilities, invalidity, and even death, as presented in this review. Similarly, these challenges apply to veterinary medicine, where increased risk of transmission in livestock would also have a financial impact that is related to livestock losses. As such, it is rather important to review the literature concerning tick-borne diseases (TBDs) and their pathogens in humans and animals, as well as their emergence in Greece.

It is noticed that current tick-related data in Greece is limited. However, the publications discussed demonstrate that there is a risk of developing preferable conditions for ticks at certain geographic areas, as well as the risk of introducing new tick-borne pathogens and new tick hosts for pathogens, and the risk of increased geographic spread in the country. Interestingly, regarding the prevalence of CCHF virus, usually transmitted by *Hy. marginatum* ticks, Papa et al. found that instead of *Hy. marginatum*, *Rh. sanguineus* and *Rh. bursa* ticks were found to be carriers of the virus [42] (Section 3.2.2). This finding could mean that pathogens change their tick vectors, and should encourage further research. Furthermore, it was found, that there are genetically diverse strains of CCHF virus circulating in the country [98], which could potentially result in the appearance of novel viral genotypes with increased resistance, pathogenicity, and fitness.

In general, many tick species find optimal living conditions in the Greek environment, as a wide variety, including resistant forms, of tick species exist (Section 2). As for all vector-borne diseases, the establishment and maintenance of endemic regions of tick-borne diseases requires environmental factors and human behavior that favor an efficient contact between an abundant burden of competent ticks, hosts with a relatively high prevalence of infection, and humans. As showcased in the present review, Greece has an abundance of ticks from a large number of species, which are widely distributed throughout the mainland country and the islands (Table 1). Importantly also, a decisive number of ticks in Greece act as vectors of multiple human and livestock pathogens, as summarized in Table 3.

**Figure 1:**
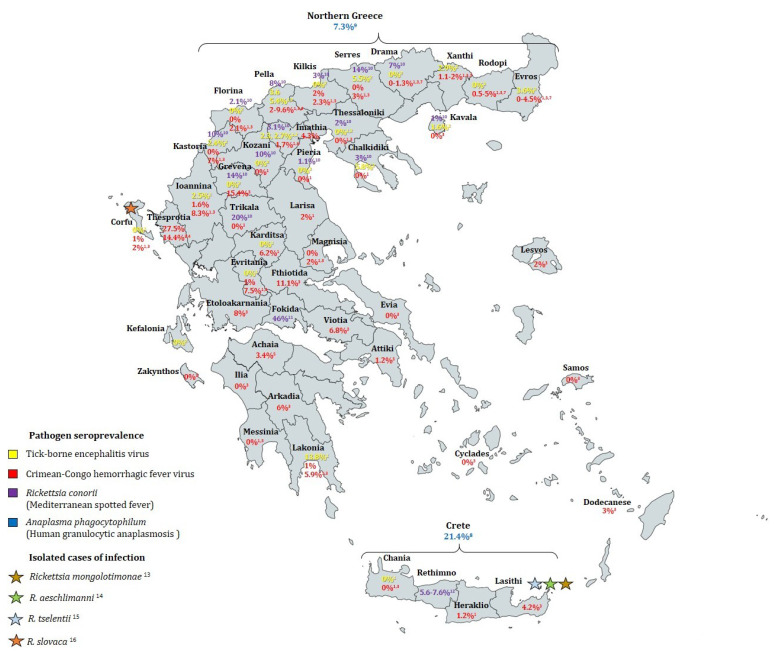
Map of the reported seroprevalence rates and isolated cases of human tick-borne diseases in different regional units of Greece, as described in the literature. ^1^ [47], ^2^ [48], ^3^ [52], ^4^ [53], ^5^ [55], ^6^ [62], ^7^ [54], ^8^ [76], ^9^ [77], ^10^ [68], ^11^ [66], ^12^ [67], ^13^ [31], ^14^ [72], ^15^ [32], ^16^ [73]. Map created with mapchart.net, accessed on 13 August 2021.

The number of publications concerning the prevalence of relevant tick-borne human diseases in Greece, such as tick-borne encephalitis (TBE) or Lyme borreliosis, is limited (Figure 1). While this could be interpreted as the circulating pathogens in Greece not constituting a major public health problem, it is also possible that a lack of clinician awareness and the absence of a nation-wide medical database leads to underdiagnosis and underestimation of case numbers.

Still, human-driven climate change and worldwide increasing mobility of people through modern traffic technologies and behavior (tourism and international trade) facilitate the transfer of pathogens and their survival in new geographic areas, where they never existed before, or where they show up again after a temporary elimination. The introduction of exotic diseases and disease vectors in Europe is primarily facilitated by globalization [164]. It is therefore expected that the occurrence of the TBE virus, Lyme disease, and other tick-borne human diseases will increase in Greece. Regular and routine country-wide tests should be undertaken to monitor the tick population and seroprevalence of tick-transmitted infections in animals and humans to map the emergence of tick-borne pathogens and diseases in the country for surveillance, control, and prevention.

Constantly updated and adjusted, effective, and rapid emergency strategies are important to contain or prevent tick-borne disease outbreaks. However, guidelines and strategies are only useful if they are communicated to the right authorities and recipients at the right time through established channels. Above all, this also includes education of the public and training of medical staff, with alerts shortly before the critical seasons.

Concerning viral tick-borne infections, Crimean-Congo hemorrhagic fever is endemic in the Balkan Peninsula, and has been reported with increasing frequency in recent years in various regions of Greece (Figure 1). Ever since the first fatal human case in 2008, there seems to be an increased circulation of CCHFV in the country (Section 3.2.3). Moreover, there is a high transmission risk of CCHFV from livestock to humans in certain areas [98]. Whether or not climatic and environmental changes have played a role in providing the favorable conditions for CCHF emergence in Greece has to be further investigated. Although it is not possible to predict its future occurrence, some models have suggested that the Mediterranean basin has become suitable for an expansion of CCHF [165], but demographic factors, farming practices, and land use change may be more important drivers [166]. It should be routine for clinicians to include CCHF in the differential diagnosis of febrile hemorrhagic syndromes, even in non-endemic regions, as the coincidence of factors benefit the emergence of new pathogens in an area, especially when neighboring countries with similar landscape are endemic. As Maltezou and Papa already proposed, it is an urgent need to identify areas of risk for CCHF and enhance surveillance [167]. The health policy should support the development of new therapies and the development of an effective and safe vaccine against CCHF.

With regard to tick-transmitted bacterial diseases (Section 4), unfortunately, reliable large-scale data concerning their prevalence in the country do not exist, because of missing evaluations, omitted case reports, absence of centralized data banks, lack of awareness, and possibly insufficient knowledge of clinical characteristics and therefore missing diagnostics. Nevertheless, a human tick-borne bacterial infectious disease with multiple reports in Greece is human granulocytic anaplasmosis (Section 4.2), a disease associated with significant morbidity and mortality. However, the mortality rates may be overestimated as no accounts of asymptomatic cases have been given [1,168]. Specifically, in Greece, HGA cases are likely to be underdiagnosed, due to the lack of clinician awareness and lack of knowledge regarding HGA epidemiology. Human cases of HGA have been described on the island of Crete [74], and seroprevalence studies suggest that the agent is circulating in additional parts of the country [77] (Figure 1). Since the first fatal case in 2017, the Hellenic Center for Disease Control and Prevention (HCDCP) issued guidance to clinicians recommending the inclusion of HGA in the differential diagnosis of patients presenting with clinical and laboratory characteristics consistent with the disease. In addition, the HCDCP has published guidelines for preventive measures against tick bites for the general population. Similar directories were given a few years earlier, in 2008, upon the diagnosis of a fatal case of CCHF in the northern part of the country [58]. Information about the case, adverse outcomes, and tick bite guidance were highly publicized in the media. Regarding the laboratory diagnosis, three laboratories were appointed to serve as national reference centers for the diagnosis of anaplasmosis and other febrile tick-borne diseases, such as ehrlichiosis, in collaboration with the veterinary laboratory at the National School of Public Health in Athens, demonstrating the high interest in the tick-transmitted disease.

It is interesting that despite the significant occurrence of borreliosis, especially Lyme borreliosis, with high public health relevance in various European countries, the data for Greece is rather poor (Section 4.3). Greece does not appear in various systematic Europe-wide literature reviews or does so only with low prevalence data. In the early review of Santino et al. about the geographical incidence of infection with *B. burgdorferi* in Europe, Greece is described with the lowest incidence of 1.1% [169]. Furthermore, no birds were found positive for the presence of *Borrelia* spp. in Greece [27], in sharp contrast with other parts of Europe, where *Borrelia* is reported to be the tick-borne pathogen with the highest prevalence in ticks removed from captured birds [163]. These findings do not necessarily indicate that *Borrelia* is absent in Greece: its presence could have been missed by the limited research, lack of updated data, or due to the locations of the studies, which were not an appropriate habitat to find *Borrelia*-infected ticks, given that *I. ricinus* ticks are primarily found in shrubs and forests with deciduous trees, while most studied areas have taken place in hot and dry semi-arid environments, which do not represent the optimum conditions for this species (Table 2). Therefore, updated information on the distribution of different *Borrelia* species in their reservoir hosts and vectors in Greece is needed, especially in the context of the main risk factors including climatic variations, certain human activities, as well as the movements of animals, people, or goods, and taking into consideration that new endemic foci of the disease may be established.

**Figure 2:**
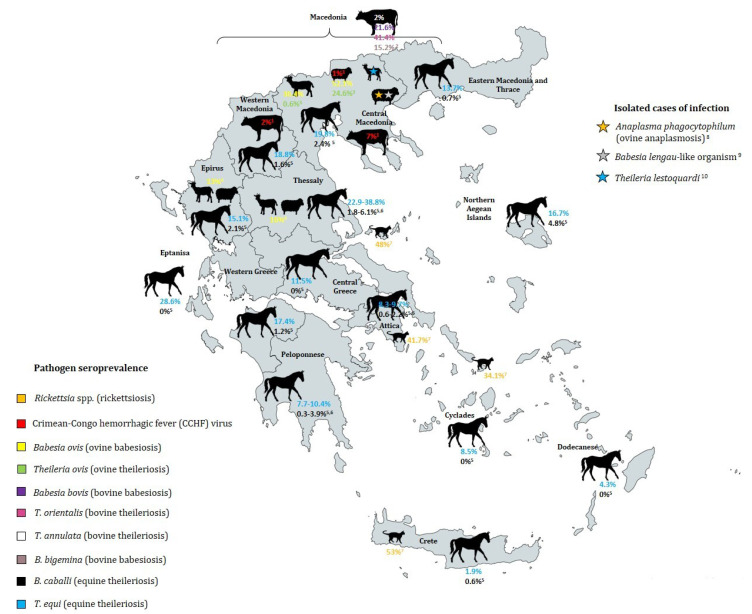
Map of the reported seroprevalence rates and isolated cases of various animal tick-borne diseases in different geographic regions of Greece, as described in the literature. ^1^ [98], ^2^ [80], ^3^ [83], ^4^ [82], ^5^ [85], ^6^ [84], ^7^ [161], ^8^ [78], ^9^ [142], ^10^ [87], ^11^ [157], ^12^ [158]. Map created with mapchart.net, accessed on 13 August 2021.

Regarding tick-borne protozoan diseases, various studies demonstrate the broad dispersal of *Babesia* species among the Greek population of small ruminants (Figure 2). Furthermore, the enzootic status of equine piroplasmosis in Greece has also been confirmed and it is supported by the occurrence of four tick vectors, namely *Rh. sanguineus*, *Rh. bursa*, *Hy. marginatum*, and *Hy. anatolicum excavatum*, which are suitable vectors for both parasitic protozoa *T. equi* and *B. caballi* in the country [170]. The wide distribution of *Rh. sanguineus* confirms that the brown dog tick can survive in different ecological niches and that environmental constraints are not very influential on the colonization success of this tick species [171]. Tick-borne diseases in farm animals can cause high economic losses relating to treatment costs, loss of performance, abortion, and death.

Both stray dogs and free-ranging cats are a major concern in Greece, and are exposed to a plethora of internal parasites and vector-borne pathogens, with some of them having the potential to infect humans (Section 5.2). Greece has been repeatedly reported as an area highly endemic for various canine vector-borne diseases caused by a range of pathogens transmitted to dogs by blood-feeding arthropods, especially ticks, but also fleas, mosquitoes, and sandflies [158]. Hence, epidemiological vigilance and appropriate control measures are crucial for the prevention and control of these infections, and to minimize the risk of transmission to humans. Specifically, routine surveillance is required in touristic areas because the movements of pets pose substantial risk in spreading pathogens because: (i) they may be introduced from enzootic to free regions when infected pets travel with their owners; and (ii) pets travelling to enzootic areas may acquire pathogens from other environments or from local animals and they are then able to introduce them into free areas when returning home. Future studies of tick-borne pathogen infection rates of companion animals belonging to tourists visiting Greece could shed light on the extent of the said risk. Crucially, many zoonotic pathogens (like *Rickettsia* spp.) infecting cats, as well as various ectoparasites (e.g., ticks and fleas), are also capable of infecting/ infesting dogs. This is of importance considering that dogs frequently travel together with their families for holidays. Therefore, control measures are fundamental for the prevention and control of zoonotic infections and external parasites in cats and dogs, via planned treatments and preventative measures with broad-spectrum and efficacious parasiticide formulations. At the same time, it is crucial to educate the general public, veterinary practitioners, and public health officials on the factual risks for diseases potentially associated with free-living cats and dogs, in order to ensure the safe movement of people and pets and to avoid direct contacts with local cats and dogs, for safe and healthy travel in the age of modern tourism.

Finally, a high level of control of infectious diseases in the country would dictate the establishment of a centralized data bank with a mission of consistently collecting all the data, in a standardized form, about pathogens, their vectors, reservoirs, related infectious disease, as well as the geographic and climatic environments. By doing so, maps and guidelines of risk factors can be developed and provided to the general population through educational initiatives, thus enhancing public awareness. With the help of a central data bank, major health problems and their development can be identified so that the health community can react adequately, consequently, effectively, and fast according to appropriate, proven, and valid (regional) protocols.

### 6.1. Perspectives in the Context of Social and Political Changes and Current Situation in Greece

An efficient initiative to explore the impact of climate change on human vulnerability to vector-borne diseases, including tick-borne diseases, and food- and water-borne diseases does not really exist in Greece, despite the dramatic increase in tourist numbers in recent years, which have reached more than three times the endogenous population during the summer periods. In addition, persisting migration and population growth and related conflicts will probably affect the distribution and prevalence of vector-borne diseases, supported by the hygienically poor living conditions for refugees in some hot spots in the country.

Historically, political instabilities have always played a critical role in the exacerbation of infectious diseases, either vector-, blood-, food-, or water-borne. Social, cultural, and political changes have major effects on the social determinants of health, mediating financial flows and human resources, and on shaping the delivery of healthcare services and disease prevention initiatives [172,173]. Economic crises and mismanagement lead to a serious shortage of skilled health care workers, laboratory facilities in universities and/or other organizations, as well as experts in the biomedical field and to the decrease of funded research. Thereby, the creation of a long-term negative impact, such as malnutrition, poor hygienic conditions, and the lack of access to medical care for the poor and socially underprivileged communities, will be aggravated and lead to increased vulnerability of the population to infectious diseases.

### 6.2. Conclusions

Compilation of historical and recent data in the context of the present review of human and animal tick-borne diseases in Greece shows that, despite circulation of tick-borne viruses, Greece is less of a hotspot for viral tick-borne diseases, and more so for tick-transmitted bacteria and protozoans, causing related diseases in humans and animals.

Demographic changes; international movements (refugees, travelers, tourists, also accompanied by pets); smuggling of wildlife; trade of animals, plants, and goods; as well as the unpredictable climatic and environmental, including land use, changes will lead to a redistribution and increase of zoonotic infections, both new and re-emerging, including TBDs. Thus, there is the need to improve epidemiological understanding, educate the public, and to establish standardized diagnostic methodologies and analytics for human and animal infections caused by tick-borne pathogens using conventional and molecular tools at a large scale in microbiological laboratories.

A comprehensive map of the geographical representation of ticks, annually updated, and their potency to carry pathogens with transmission risk to humans and animals would be an essential step towards prediction, control, and prevention models, strategies, and campaigns. In parallel, programs should be developed to calculate the probability of the presence of a pathogen in terms of predicting or forecasting tick-borne diseases, especially considering that chemical or pharmaceutical preventive and therapeutic measures are limited. Systematic investigations for all relevant tick-borne pathogens and diseases for humans and animals of agricultural importance are still required, helping to better understand the interactions of integrated strategies for the management of the tick population and the distribution and control of TBDs.

## Figures and Tables

**Table 1:** Hard tick species (*Ixodidae)* found in Greece and their associated pathogens, hosts, and regions of emergence.

Tick Species	Region	Host	Associated Pathogen(s)	References
*Amblyomma variegatum*	Macedonia	Goats	No pathogens reported	[15]
*Dermacentor marginatus*	Northern Greece (Macedonia), Cephalonia Island, Lesvos Island, Western and Central Greece	Cattle, sheep, goats, dogs, humans	CCHF virus, *Rickettsia* spp., *Anaplasma* spp., *C. burnetii*, *Babesia* sp., *Theileria* sp., *T. annulata*, *T. bufeli/orientalis*, *T. ovis*, *T. lestoquardi*, *Coxiella*-like endosymbionts	[16,17,18,19,20,21,22,23]
*Haemaphysalis impeltatum*	Central Greece	Sheep, goats, cattle	No pathogens reported	[19,20]
*Haemaphysalis inermis*	Northern Greece (Macedonia)	Sheep, goats, cattle, dogs	No pathogens reported	[15]
*Haemaphysalis parva*	Northern Greece (Macedonia, Thrace), Lesvos Island, Thessaly, Arta, Etoloakarnania, Evritania, Fokida, Ftiotida, Preveza	Cattle, sheep, goats, dogs	CCHF virus, *Rickettsia* spp., *R. hoogstraalii, Anaplasma* spp., *Hepatozoon canis, H. felis,* phleboviruses, *C. burnetii, Babesia* sp., *B. ovis*, *B. crassa, Theileria* sp., *T. annulata, T. ovis, T. lestoquardi*	[15,20,22,23,24,25]
*Haemaphysalis punctata*	Northern Greece (Macedonia), Cephalonia Island, Lesvos Island, Arta, Crete	Sheep, goats, cattle, dogs	No pathogens reported	[15,17,20,22,23,25,26]
*Haemaphysalis sulcata*	Northern Greece (Macedonia), Cephalonia Island, Arta, Etoloakarnania, Fokida, Preveza, Thessaloniki	Cattle, sheep, goats	*Rickettsia spp., Anaplasma* spp., *C. burnetii*, *Babesia* sp., *B. divergens, Theileria* sp., *T. ovis*	[15,17,20,22,23]
*Hyalomma aegyptium*	Serres region in Northern Greece (Lailias mountain region)	Wild birds, tortoises	*Rickettsia* spp., *R. aeschlimannii*, *Borrelia turcica*	[27,28,29]
*Hyalomma anatolicum anatolicum*	Northern Greece	Sheep, goats, cattle	No pathogens reported	[19,30]
*Hyalomma anatolicum excavatum*	Northern Greece (Macedonia), Cephalonia Island, Crete	Sheep, goats, cattle, horses, humans	*Rickettsia aeschlimannii*, *R. sibirica mongolotimonae*	[15,17,19,20,31,32],
*Hyalomma concinna*	Northern Greece (Thrace), Thessaly	Dogs	*Hepatozoon canis*	[24]
*Hyalomma detritum scupense*	Northern Greece (Macedonia, Thrace)	Dogs, cattle	No pathogens reported	[15,24]
*Hyalomma dromedarii*	Northern Greece	Sheep, goats, cattle	No pathogens reported	[19,20,33]
*Hyalomma marginatum marginatum (*syn. *Hy. plumbeum plumbeum)*	Northern Greece (Macedonia, Thrace), Cephalonia Island	Sheep, goats, horses, cattle, dogs, humanswild birds	CCHF virus, *T. equi*, *Rickettsia* spp.	[15,17,18,19,20,27,33,34,35,36]
*Hyalomma marginatum rufipes*	Northern Greece (Macedonia, Thrace), Antikythira Island	Sheep, goats, cattle, humans, wild birds	*Rickettsia* spp.	[15,19,20,36,37,38]
*Hyalomma marginatum turanicum*	Northern Greece (Macedonia)	Cattle	No pathogens reported	[15,19]
*Ixodes* *acuminatus*	Northern Greece	Wild birds	No pathogens reported	[27]
*Ixodes* *frontalis*	Northern Greece	Wild birds	*Rickettsia* spp.	[27]
*Ixodes gibbosus*	Northern Greece (Macedonia), Komotini, Limnos Island, Cephalonia Island	Sheep, goats, cattle, horses, humans	*Rickettsia* spp., *Anaplasma* spp., *C. burnetii, Theileria* sp., *T. lestoquardi*	[15,16,17,18,20,22,23]
*Ixodes hexagonus*	Northern Greece (Macedonia)	Dogs	No pathogens reported	[15]
*Ixodes ricinus*	Chalkidiki, Rodopi, Macedonia, Thrace, Kastoria	Sheep, goats, dogs, cattle, humans	TBE virus, *Rickettsia monacensis*, *R. massiliae*, *Anaplasma phagocytophilum*, *A. platys, B. divergens, Coxiella-like endosymbionts*	[15,18,19,20,21,24,35,38,39]
*Rhipicephalus (Boophilus) annulatus*	Northern Greece (Macedonia, Thrace), Komotini, Chalkidiki	Cattle, sheep, goats, humans	No pathogens reported	[15,18,19,35,36,40]
*Rhipicephalus bursa*	Kastoria, Fokida, Macedonia, Thrace, Rodopi, Cephalonia Island	Sheep, goats, cattle, dogs, horses, humans	*Rickettsia monacensis*, *R. massiliae*, *R. slovaca*, *Candidatus R. barbariae, R. rhipicephali, Anaplasma phagocytophilum, A. platys*, CCHF virus, *Theileria ovis, Coxiella-like* endosymbionts	[15,16,17,18,19,20,21,22,23,24,33,38,40]
*Rhipicephalus camicasi*	Northern Greece	Sheep, goats, cattle	No pathogens reported	[19,33]
*Rhipicephalus sanguineus*	Country-wide	Sheep, goats, dogs, cats, cattle, humans	CCHF virus, *Rickettsia *spp.* (R. conorii, R. aeschlimannii, R. africae, R. massiliae, R. rhipicephali), Ehrlichia canis, Hepatozoon canis, H. felis, Anaplasma* spp., *A. platys, Coxiella burnetii, Babesia* sp., *B. bigemina, B. ovis, B. caballi, Theileria* sp., *T. annulata, T. bufeli/orientalis, T. ovis, T. lestoquardi*	[15,17,18,19,20,21,22,23,24,34,38,41,42]
*Rhipicephalus turanicus*	Northern Greece (Macedonia, Thrace), Cephalonia Island	Sheep, goats, cattle, dogs, horses, humans	*Rickettsia aeschlimannii, R. massiliae, Hepatozoon canis*	[15,17,18,19,24]

**Table 2:** Summary of the key findings of published studies on the prevalence of hard tick species parasitizing animals and/or humans in Greece.

Study	Sampling Period	Geographic Region	Animals Sampled	Sampling Strategy	Total Ticks Sampled	Tick Species (Relative Species Distribution)
**[15]**	Mar–Jun and Nov–Dec 1983, Apr–May and Sep–Oct 1985, Apr–Jul and Oct–Nov 1986	Northern Greece (Macedonia, 64 localities)	Cattle (602)	Tick collection from grazing herds of 5–20 animals	5137 ticks (16 species)	*Rhipicephalus bursa* (29.38%); *Boophilus annulatus* (23.83%); *Ixodes ricinus* (10.47%); *Rh. turanicus* (8.92%); *Hyalomma m. marginatum* (7.96%); *Haemaphysalis inermis* (7.46%); *l. gibbosus* (7.40%); *H. anatolicum excavatum* (3.99%); *H. detritum scupense* (3.78%); *H. punctata* (1.77%); *Dermacentor marginatus* (0.93%); *H. parva* (0.56%); *H. sulcata* (0.12%); *H. marginatum rufipes* (0.04%); *Rh. sannguineus* (0.02%); *H. marginatum turanicum* (0.02%)
			Sheep (721)		2265 ticks (12 species)	*Rhipicephalus bursa* (47.95%); *Rh. turanicus* (18.59%); *H. sulcata* (14.79%); *H. punctata* (4.77%)*; Dermacentor marginatus* (4.72%); *Haemaphysalis inermis* (4.19%); *Ixodes ricinus* (2.25%); *l. gibbosus* (1.99%); *H. parva* (0.53%); *Rh. sannguineus* (0.09%); *Hy. anatolicum excavatum* (0.09%); *Hyalomma m. marginatum* (0.04%)
			Goats (487)		3231 ticks (12 species)	*Rhipicephalus bursa* (49.98%)*; l. gibbosus* (23.03%); *H. sulcata* (9.97%); *Rh. turanicus* (6.47%)*; Ixodes ricinus* (3.65%); *Haemaphysalis inermis* (2.88%); *Dermacentor marginatus* (2.38%); *H. punctata* (1.39%); *H. parva* (0.12%); *Hyalomma m. marginatum* (0.06%); *Hy. anatolicum excavatum* (0.03%); *Amblyomma variegatum* (0.03%)
			Dogs (70)		987 ticks (6 species)	*Rh. sannguineus* (60.49%); *Rh. turanicus* (29.69%); *Ixodes ricinus* (8.41%); *Rhipicephalus bursa* (0.91%); *I. hexagonus* (0.30%); *Haemaphysalis punctata* (0.20%)
**[43]**	1996–1997	Thessaloniki	Dogs (249)	Dogs admitted to local veterinary hospital and private practices	2812 ticks (2 species)	*Rh. sanguineus* (89.30%); *Rh. turanicus* (5.55%); *Rhipicephalus* spp. nymphs and larvae (5.16%)
**[44]**	May–Aug 1998	Northern Greece (3 villages in Fokida region)	Goats, sheep, and dogs (not specified)	Not specified	439 ticks (3 species)	*Rhipicephalus sanguineus* (47.15%); *Rh. bursa* (31.44%); *Rh. turanicus* (21.41%)
**[17]**	Apr–Jul 1998, May–Jun and Oct 1999	Corfu Island (32 localities)	Cattle (not specified)	Not specified	145 ticks (5 species)	*Hyalomma marginatum marginatum* (31.03%); *Hy. anatolicum excavatum* (25.52%)*; Rhipicephalus bursa* (20.69%); *Rh. turanicus* (20.00%); *Ixodes gibbosus* (2.76%)
			Sheep (not specified)		524 ticks (7 species)	*Rh. turanicus* (38.74%); *Dermacentor marginatus* (29.39%); *Rhipicephalus bursa* (11.45%), *Hy. marginatum marginatum* (11.07%); *Hyalomma (Hy.) anatolicum excavatum* (4.58%); *Haemaphysalis (Ha.) punctata* (4.39%); *Ixodes gibbosus* (0.38%)
			Goats (not specified)		796 ticks (8 species)	*Rh. turanicus* (34.92%); *Dermacentor marginatus* (29.65%); *Rhipicephalus bursa* (29.15%); *Ixodes gibbosus* (3.02%); *Haemaphysalis sulcata* (1.38%); *Ha. punctata* (0.75%)*, Hyalomma marginatum marginatum* (0.63%); *Hy. anatolicum excavatum* (0.50%)
			Horses (not specified)		86 ticks (5 species)	*Rh. turanicus* (58.14%); *Hy. marginatum marginatum* (19.77%); *Hy. anatolicum excavatum* (17.44%); *Rhipicephalus bursa* (2.33%); *Ixodes gibbosus* (2.33%)
			Dogs (not specified)		297 ticks (3 species)	*Rh. sanguineus* (96.97%); *Hy. marginatum marginatum* (1.68%); *Rh. turanicus* (1.35%)
**[45]**	Apr–Jul and Sep–Dec of 2003–2006	Northern Greece (11 prefectures of Macedonia and Thrace)	Sheep and goats (not specified)	Animals grazing permanently in the countryside	3144 ticks (7 species)	*Ixodes ricinus* (46.06%); *Rhipicephalus bursa* (19.78%); *Hyalomma marginatum* (12.82%); *Rh. sanguineus* (6.93%); *Rh. turanicus* (5.66%); *Boophilus annulatus* (4.58%); *I. gibbosus* (4.17%)
**[40]**	Nov and Jun 2004–2006	4 localities of Halkidiki prefecture, Northern Greece	Goats, sheep, and dogs (not specified)	Not specified	703 ticks (6 species)	*Ixodes ricinus* (50.50%); *Hyalomma marginatum* (18.92%); *Rhipicephalus bursa* (14.37%); *Rh. sanguineus* (10.81%); *Rh. turanicus* (3.56%); *Boophilus annulatus* (1.85%)
**[36]**	Jun–Sep 2008	North-eastern Greece (Komotini, Alexandroupoli)	Humans (not specified, >300)	Individuals with tick bites visiting 2 hospitals in the region	519 ticks (9 species)	*Rhipicephalus sanguineus* (81.50%); *Rh. turanicus* (5.20%); *Hyalomma marginatum* (5.20%); *Rh. bursa* (2.70%); *Hy. rufipes* (1.93%); *Ixodes gibbosus* (1.16%); *Rh. annulatus* (0.96%); *I. rinicus* (0.96%); *Hy. anatolicum* (0.39%)
**[33]**	Apr–Jun 2012	Northern Greece (30 villages in Kastoria, Rodopi, Xanthi, Kavala and Chalkidiki)	Sheep and goats (not specified)	Not specified	95 ticks (7 species)	*Rhipicephalus sanguineus* (55.79%); *Rh. bursa* (29.47%); *Rh. turanicus* (9.47%); *Rh. camicasi* (2.11%); *Hyalomma marginatum* (1.05%); *Hy. dromedarii* (1.05%); *Dermacentor marginatus* (1.05%)
**[19]**	Mar–Oct 2012–2013	Country-wide (26 mainland prefectures and 5 Aegean islands)	Cattle (not specified)	Not specified	142 ticks (11 species)	*Hy. dromedarii* (47.18%); *Hy. marginatum* (16.90%); *Rh. sanguineus* (10.56%); *Hy. excavatum* (5.63%); *Rh. bursa* (4.23%); *Rh. turanicus* (4.23%); *Rh. camicasi* (3.52%); *Hy. rufipes* (2.82%); *Hy. anatolicum* (2.11%); *Hy. turanicum* (2.11%); *Hy. impeltatum* (0.70%)
			Humans (not specified)	Infected individuals who visited hospitals	701 ticks (10 species)	*Rh. sanguineus* (80.17%); *Rh. turanicus* (4.85%); *Hy. marginatum* (4.28%); *Rh. bursa* (3.28%); *Rhipicephalus* nymphs (2.85%); *Hy. rufipes* (1.57%); *Ixodes ricinus* (0.85%); *I. gibosus* (0.85%); *Rh. annulatus* (0.71%); *Hy. excavatum* (0.28%); *Dermacentor marginatus* (0.28%)
**[20]**			Sheep (1201)	Random sampling of ~10 animals from each of 309 farms	1339 ticks (13 species)	*Rhipicephalus sanguineus* (64.15%); *Rh. bursa* (26.59%); *Dermacentor marginatus* (2.39%); *Hy. dromedarii* (2.32%); *Haemaphysalis parva* (1.27%); *Ha. sulcata* (1.12%); *I. gibbosus* (0.82%); *Hy. excavatum* (0.52%); *Hy. rufipes* (0.30%); *Hyalomma marginatum* (0.22%); *Ha. punctata* (0.07%); *Ixodes ricinus* (0.07%); *Hy. impeltatum* (0.07%); *Rhipicephalus* nymph (0.07%)
			Goats (681)		769 ticks (6 species)	*Rhipicephalus sanguineus* (65.93%); *Rh. bursa* (24.71%); *Dermacentor marginatus* (7.15%); *Haemaphysalis parva* (1.04%); *Ha. sulcata* (0.91%); *Hyalomma marginatum* (0.26%)
**[24]**	May–Aug 2015	6 provinces of mainland Greece (Larisa, Athens, Corinth, Thessaloniki, Xanthi, Alexandroupo-li)	Dogs (310)	Domestic dogs from rural areas, (shelters, temporary kennels, indoor environments, or hospitals)	757 ticks (7 species)	*Rhipicephalus sanguineus* (s.l.) (70.15%); *Haemaphysalis parva* (14.66%); *Rh. turanicus* (11.36%); *H. concinna* (2.38%); *Ixodes ricinus* (0.26%); *Hyalomma scupense* (0.13%); *Rh. bursa* (0.13%)
**[21]**	Feb 2015–May 2016	Northern Greece (5 prefectures)	Goats (28)	Not specified	64 ticks (5 species)	*Rh. bursa* (57.81%)*; D. marginatus* (17.19%)*; I. ricinus* (15.63%); *Rh. sanguineus* (4.69%)*; Haemaphysalis parva* (3.13%)*; Rhipicephalus spp. nymph* (1.56%)
**[23]**	Mar–Oct 2014	Central and Northern Greece (14 mainland prefectures)	Sheep (not specified)	Random sampling of 10 animals in each farm	64 ticks (6 species)	*Dermacentor marginatus* (26.56%); *Haemaphysalis parva* (26.56%); *H. sulcata* (23.44%); *Ixodes gibbosus* (20.31%); *H. punctata* (1.56%), *Rhipicephalus sanguineus* (1.56%)
			Goats (not specified)		42 ticks (5 species)	*Dermacentor marginatus* (54.76%); *Haemaphysalis parva* (19.05%); *H. sulcata* (6.67%); *Rhipicephalus sanguineus* (7.14%); *Rh. bursa* (2.38%)
			Dogs (not specified)	Dogs presented to veterinary clinics	73 ticks (1 species)	*Rh. sanguineus s.l.* (100%)
**[46]**	Mar–Oct 2014	13 prefectures of Central and Northern mainland Greece, and Limnos Island	Sheep (not specified)	Not specified	63 ticks (5 species)	*Dermacentor marginatus* (26.98%); *Haemaphysalis parva* (26.98%); *H. sulcata* (23.81%); *Ixodes gibosus* (20.63%); *Rhipicephalus sanguineus* (1.59%)
			Goats (not specified)		43 ticks (6 species)	*Dermacentor marginatus* (53.49%); *Haemaphysalis parva* (18.60%); *H. sulcata* (16.28%); *Rhipicephalus sanguineus* (6.98%); *Rh. bursa* (2.33%); *H. punctata* (2.33%)
			Dogs (not specified)		78 ticks (1 species)	*Rhipicephalus sanguineus* (93.59%); *Rhipicephalus* sp. (6.41%)

**Table 3:** Important tick-borne pathogens, associated human and domestic animal diseases, and their prevalence in Greece.

Pathogen	Disease	Tick Vectors (Transmitters)	Clinical Symptoms	Prevalence in Greece	Geographic Areas of High Prevalence	Season/Climate of High Prevalence	References
Tick-borne encephalitis (TBE) virus–Greek goat encephalitis virus (GGEV)	Tick-borne encephalitis	*I. ricinus* (European subtype)	Disorder of the nervous system with symptoms of meningitis and encephalitis	1.7% general prevalence (1981–1988) [47]	2.1% in Northern Greece and Kefalonia Island, (Chalkidiki, Pella and Serres > 5.3%) (2003–2005) [48] 13.8% in Laconia (1981–1988) [47]	October to November in Northern Greece, high relative humidity	[49,50]
Crimean-Congo hemorrhagic fever (CCHF) virus	Crimean-Congo hemorrhagic fever	*Hyalomma marginatum* (main vector), *Dermacentor marginatus, Rhipicephalus sanguineus,* *Rh. bursa* (vector of Greek strain AP92), *Haemaphysalis parva*	Affects whole body by hemorrhage, kidney failure, pulmonary distress and shock, mortality of ~30%. First fatal case in Rodopi in June 2008 [51]	Country-wide seroprevalence 4.2% (2009–2010) [52] 1% nation-wide (1981–1988) [47]	14.4% in Thesprotia (2010–2012) [53] 9.6% in Pella, 6.2% in Karditsa (1981–1988) [47] 6% in Kastoria (2012) [33] 5% in Rodopi, 4.5% in Evros (2008–2009) [54] 3.4% in Achaia [55]	Altitude >400 m, special soil conditions, May to August, increased *R. bursa* activity with higher temperatures and lower humidity	[22,30,42,56,57,58,59,60,61,62]
*Rickettsia massiliae*	Rickettsiosis of spotted fever group (SFG)	*Rhipicephalus sanguineus, Rh. turanicus, Rh. bursa, Ixodes ricinus*	Fever, rash, headache, severe myalgia, weakness	No data	No data	*Rhipicephalus sanguineus* common in meso-Mediterranean bioclimatic zone, active during spring/early summer	[17,21,38,44,63]
*Rickettsia conorii*	Mediterranean Spotted Fever (MSF)	*Rhipicephalus sanguineus*	High fever, myalgia, lymphadeno-pathy, headache, maculopapular rash, neurological complications	Fatal case in Northeastern Greece in 2008 [64]	Crete (1985–1987): 5.6% and 2.2% in a village, 0.5% and 0% in another [65] 46% in Fokida (1991) [66] Rethymno prefecture in Crete: 5.6% (1985), 7.6% (1998) [67] 7.9% in Northern Greece: 20% in Trikala, 14% in Grevena and Serres (2000) [68]	Adult *Rh. sanguineus* in May, larvae and nymphs active during summer	[26,69,70,71]
*Rickettsia sibirica mongolotimonae*	Lymphangitis- associated Rickettsiosis (LAR)	*Hyalomma anatolicum* * excavatum*	Fever, maculopapular rash, enlarged regional lymph nodes, lymphangitis	First reported human case in Crete in 2002, [31]	No data	No data	[32]
*Rickettsia aeschlimannii*	Tick-borne lymphadeno-pathy (TIBOLA)	*Rhipicephalus turanicus, Rh. sanguineus, H. anatolicum excavatum*	Non-specific, inoculation eschar, fever, generalized maculopapular rash	First reported human case in Europe in Crete [72]	No data	No data	[17,32,38]
*Rickettsia slovaca*	Tick-borne lymphadeno-pathy (TIBOLA)	*Rhipicephalus bursa*	Enlarged regional lymph nodes, fever and malaise, maculopapular rash	First reported human case in Corfu in 2011 [73]	No data	May–August: *Rh. bursa* activity increased with high temperature and low humidity	[40]
*Anaplasma phagocytophilum*	Human granulocytic anaplasmosis (ehrlichiosis), ovine anaplasmosis	*Ixodes ricinus*, *Rhipicephalus bursa*	Fever, chills, maculopapular rash, headache, malaise, splenomegaly, Cervical lymph-adenopathy, gastrointestinal disturbances	First reported case in Crete [74], first fatal case in Athens [75]	21.4% in Crete (2005–2006) [76] 7.3% in Northern Greece (2000) [77]	Peak tick activity in the summer	[39,78]
*Borrelia burgdorferi*	Lyme disease	*Ixodes ricinus* complex	Multisystemic manifestation: Erythema migrans, neuroborreliosis with peripheral or central affection, joints (arthritis), skin (acro-dermatitis chronica atrophicans), *Borrelia* lymphocytoma, cardiovascular, renal (glomerulo-nephritis), chronification possible	3.27% IgG positive by enzyme immunoessay and 0.27% IgG pos. Western Blot in 1100 Greek male Navy recruits	No data	No data	[79]
*Borrelia turcica*	Phylogenetic tree and goeBURST analysis showed differences to Lyme disease (LD) and relapsing fever (RF) species	*Hyalomma aegyptium*	Unknown pathogenicity for humans	131 (38.6%) of 339 ticks *Borrelia* positive (conventional flaB PCR) (2000), and 58 (64.4%) (23S locus qPCR) of 90 screened ticks (of 358 collected in total) *Borrelia* positive	Location of tick collection in Serres region in Northern Greece (Lailias mountain region) (2000 and 2017)	Tick collection time during spring (May–June) (2000 and 2017)	[28,29]
*Babesia bovis*	Bovine babesiosis (most common babesiosis in Greece)	*Boophilus annulatus* (proposed)	Acute and very severe infection (high fever, anemia anorexia)	No data	21.6% of 602 cattle in Macedonia (1984–1986) [80]	No data	[81]
*Babesia bigemina*	Bovine babesiosis (rare cases in Greece)	*Boophilus annulatus* (proposed)	Less severe than *B. bovis* infection	No data	15.2% of 602 cattle in Macedonia (1984–1986) [80]	No data	[81]
*Babesia ovis*	Ovine babesiosis	*Rhipicephalus bursa, Ixodes scapularis* (proposed)	Apathy, fever, anemia, jaundice, haemoglobinuria, death	No data	Thessaly and Epirus: 17.4% of 124 sheep, 9.4% of 32 goats (2002) [82] Macedonia: 52.1% of 721sheep, 36.4% of 487 goats (1984–1985) [83]	No data	
*Babesia caballi*	Equine piroplasmosis	*Rhipicephalus* *sanguineus*	Non-specific haemolytic conditions (fever, anaemia, jaundice)	2.2% of 544 equids in the mainland (2007–2008) [84] 1.3% of 7872 equines country-wide (2001–2008) [85]	6.1% of 49 equids in Thessaly [84] Clusters of both *Theileria* and *Babesia* spp. in Macedonia and Thessaly [86]	No data	[34]
*Theileria ovis*	Benign theileriosis of small ruminants	*Rhipicephalus bursa*	Non-pathogenic	No data	Macedonia: 24.6% of 721 sheep, 0.6% of 487 goats (1984–1985) [83]	No data	[87]
*Theileria orientalis*	Bovine theileriosis	*Rh. bursa*, *H. punctata* (proposed)	Non-pathogenic	No data	41.4% of 602 cattle in Macedonia (1984–1986) [80]	No data	[83]
*Theileria annulata*	Mediterranean bovine theileriosis	*Hyalomma d. detritum* (proposed)	No clinical symptoms	No data	2% of 602 cattle in Macedonia (1984–1986) [80]	Warm Mediterranean maquis	[83]
*Theileria equi* and *T. equi*-like genotype	Equine piroplasmosis	*Hyalomma marginatum*	Non-specific haemolytic conditions (fever, anaemia, jaundice), more severe than *B. caballi*	11% of 544 equids in mainland Greece (2007–2008) [84] 12.9% of 7872 equines country-wide (2001–2008) [85]	38.8% of 49 equids in Thessaly [84]	Occurrence most predominantly in forest type land cover class	[34]

**Table 4:** Summary of the key findings of published studies on the seroprevalence of TBDs in humans in Greece.

	Citation	Sampling Period	Geographic Region	Humans Tested	Sampling Strategy	Detection Method	Positive Rate (Seroprevalence)
**TBE**	[92]	1958	Athens	56	Not specified	Hemagglutination-inhibition (HI) and neutralization tests	1.8%
	[47]	1981–1988	Mainland Greece, islands of Corfu and Crete	475	Apparently healthy individuals (farmers, wood cutters & shepherds)	Indirect immunofluorescence (IFA)	Overall 1.7%. Regions of non-zero seroprevalence: Laconia (13.8%), Pella (3.57%), Ioannina (2.47%), Imathia (2.27%)
	[48]	2003–2005	Northern Greece (all prefectures) and Corfu Island	921	Apparently healthy permanent residents of urban and rural areas	Serum samples: IFA and ELISA (samples considered positive if positive by both). Cerebrospinal fluid samples: 2 RT-nPCR protocols	Overall 2.06%. Regions of non-zero seroprevalence: Chalkidiki (5.82%), Evros (3.57%), Imathia (2.7%), Kastoria (2.43%), Kavala (1.56%), Pella (5.35%), Serres (5.45%), Xanthi (2.94%)
		Jan 2003–May 2006		302	Hospital patients with CNS infection		0%
**CCHF**	[57]	1980–1981	Imathia (Central Macedonia)	65	Not specified	IFA and HI	6.2%
	[30]	Apr 1987–Apr 1988	Northern Greece (Evros, Rodopi, Halkidiki, Ioannina)	597	Not specified	IFA and ELISA	1.2%
	[47]	1981–1988	Mainland Greece, islands of Corfu and Crete	3388	Apparently healthy individuals (farmers, wood cutters & shepherds)	IFA	Overall 1%. Regions of non-zero seroprevalence: Rodopi (0.5%), Xanthi (1.2%), Kilkis (2%), Pella (9.6%), Imathia (4.3%), Evritania (1%), Larisa (2%), Karditsa (6.2%), Ioannina (1.6%), Laconia (1%), Iraklio (1.2%). Corfu (1%)
	[52]	Jun 2009–Dec 2010	Country-wide (28 prefectures)	1611	Random sampling of patients referred to primary health-care centers for blood testing with no signs of infectious disease	ELISA	Overall 4.2%. Regions of non-zero seroprevalence: Thesprotia (27.5%), Grevena (15.4%), Fthiotida (11.1%), Etoloakarnania (8.0%), Ioannina (8.3%), Evritania (7.5%), Kastoria (7.0%), Viotia (6.8%), Arkadia (6.0%), Lakonia (5.9%), Rodopi (4.95%), Evros (4.49%), Lasithi (4.2%), Dodecanese (3.0%), Serres (3.0%), Lesbos (2%), Magnesia (2.0%), Kilkis (2.3%), Corfu (2.0%), Florina (2.1%), Pella (2.0%), Xanthi (1.99%), Drama (1.34%), Attiki (1.2%)
	[53]	2010–2012	Thesprotia prefecture	166	Permanent residents randomly selected among persons who visited local health centers	ELISA	14.4%
	[55]	Mar–Jul 2012	Achaia prefecture	207	Random sampling of patients referred to primary health-care centers for blood testing with no signs of infectious disease	ELISA, confirmation by IFA	3.4%
	[62]	2010–2011	Imathia and Pella prefectures	277	Random selection of residents who visited local health centers for testing (not for infectious diseases) or blood donation	ELISA, confirmation by IFA	2.2%
				51	People belonging in groups of risk for CCHFV (19 slaughterhouse workers, 32 hunters)		3.1% in hunters, 5.3% in slaughterhouse workers
	[33]	Sep–Oct 2012	Kastoria regional unit	100	People who visited local health center with no signs of infectious disease	ELISA, confirmation by IFA	6%
	[54]	Nov 2008–Apr 2009	Northern Greece (Drama, Kavala, Xanthi, Rodopi, and Evros prefectures)	1178	Random sampling of patients referred to primary health-care centers for blood testing, regardless of reason for testing and CCHFV risk factors	ELISA	Overall 3.14%. Regions of non-zero seroprevalence: Rodopi 4.95%, Evros 4.49%, Drama 1.34%, Xanthi 1.09%
**MSF**	[65]	1985 and 1987	2 villages in Crete (Iraklio and Rethimno prefectures)	419	Random selection of 97 households	IFA	Tymbaki village: 5.6%, 2.2% after 2 years Anogia village 2: 0.5%, 0% after 2 years
	[67]	Oct 1998	Anogia village, Rethimno prefecture, Crete	238	Random selection of 84 households	IFA	7.6%
	[66]	1991	3 rural villages in Fokida province	254	Not specified	IFA and Western blot	45.3%
	[68]	Apr–Oct 2000	Northern Greece (14 prefectures)	1584	Random selection of samples collected by prefecture hospitals	IFA	Overall 7.9%. Regions of non-zero seroprevalence: Drama (7%), Florina (2.1%), Grevena (14%), Imathia (3.1%), Kastoria (10%), Kavala (1%), Khalkidiki (7.3%), Kilkis (3%), Kozani (10%), Pella (8%), Pieria (1.1%), Serres (14%), Thessaloniki (2%), Trikala (20%)
**HGA**	[77]	Apr–Oct 2000	Northern Greece	300	Healthy individuals hospitalized for routine blood tests	IFA	7.3%
	[76]	Aug 2005–Aug 2006	Crete	496	Healthy blood donors	IFA	21.4%
**LB**	[79]	Sep 1997–Nov 1998	Country-wide	1100	Randomly selected healthy Greek Navy recruits (18–25 years old)	ELISA	3.27%

TBE = Tick-borne encephalitis, CCHF = Crimean-Congo hemorrhagic fever, MSF = Mediterranean spotted fever, LB = Lyme borreliosis.
